# Metabolite profiling of chickpea (*Cicer arietinum*) in response to necrotrophic fungus *Ascochyta rabiei*

**DOI:** 10.3389/fpls.2024.1427688

**Published:** 2024-08-13

**Authors:** Rosy Raman, Stephen Morris, Niharika Sharma, Kristy Hobson, Kevin Moore

**Affiliations:** ^1^ Department of Primary Industry Research and Development, Wagga Wagga Agricultural Institute, Wagga Wagga, New South Wales, Australia; ^2^ Department of Primary Industry Research and Development, Wollongbar Primary Industries Institute, Wollongbar, New South Wales, Australia; ^3^ Department of Primary Industry Research and Development, Orange Agricultural Institute, Orange, New South Wales, Australia; ^4^ Department of Primary Industry Research and Development, Tamworth Agricultural Institute, Tamworth, New South Wales, Australia

**Keywords:** chickpea, Ascochyta blight, host-pathogen interaction, metabolomics, LC-MS

## Abstract

**Introduction:**

Ascochyta blight (AB) caused by the necrotrophic fungus *Ascochyta rabiei* is one of the most significant diseases that limit the production of chickpea. Understanding the metabolic mechanisms underlying chickpea-*A.rabiei* interactions will provide important clues to develop novel approaches to manage this disease.

**Methods:**

We performed metabolite profiling of the aerial tissue (leaf and stem) of two chickpea accessions comprising a moderately resistant breeding line (CICA1841) and a highly susceptible cultivar (Kyabra) in response to one of the highly aggressive Australian *A. rabiei* isolates TR9571 via non-targeted metabolomics analysis using liquid chromatography-mass spectrometry.

**Results:**

The results revealed resistance and susceptibility-associated constitutive metabolites for example the moderately resistant breeding line had a higher mass abundance of ferulic acid while the levels of catechins, phthalic acid, and nicotinic acid were high in the susceptible cultivar. Further, the host-pathogen interaction resulted in the altered levels of various metabolites (induced and suppressed), especially in the susceptible cultivar revealing a possible reason for susceptibility against *A.r abiei*. Noticeably, the mass abundance of salicylic acid was induced in the aerial tissue of the susceptible cultivar after fungus colonization, while methyl jasmonate (MeJA) was suppressed, elucidating the key role of phytohormones in chickpea-*A. rabiei* interaction. Many differential metabolites in flavonoid biosynthesis, phenylalanine, Aminoacyl-tRNA biosynthesis, pentose and glucuronate interconversions, arginine biosynthesis, valine, leucine, and isoleucine biosynthesis, and alanine, aspartate, and glutamate metabolism pathways were up- and down-regulated showing the involvement of these metabolic pathways in chickpea-*A. rabiei* interaction.

**Discussion:**

Taken together, this study highlights the chickpea − *A. rabiei* interaction at a metabolite level and shows how *A. rabiei* differentially alters the metabolite profile of moderately resistant and susceptible chickpea accessions and is probably exploiting the chickpea defense pathways in its favour.

## Introduction

The global production of chickpea (*Cicer arietinum L.*), a high-value legume crop for human consumption is under constant threat from Ascochyta blight (AB) caused by a necrotrophic fungus, *A. rabiei* (Singh et al., 2022; Sharma et al., 2023). In Australia, the multimillion-dollar chickpea industry spends about $34.9 million on disease control with $4.8 million going into yield losses (Vaghefi et al., 2024). *A. rabiei* can survive and spread through infected seeds, stubble, and volunteers. Under ideal temperatures (15 to 25°C) and humid conditions (3 to 10 hours of leaf wetness), the disease develops quickly, and infection can occur on all aerial parts of the plant: leaves, petiole, stem, pods, and seeds (Wilson, 2021). However, girdling from stem lesions is most damaging to the crop causing stem breakages leading to plant death and extensive yield loss (Singh et al., 2022; Morcuende et al., 2024).

Ascochyta blight can be effectively managed by integrated disease control strategies based on rotation, fungicide regime, and growing resistant cultivars (Gan et al., 2006; Fanning et al., 2022; Deokar et al., 2024). The majority of fungicides are preventative with very little curative effect, and timely and repeated applications (up to 12) of fungicides are required for effective disease control in Australia (Saloti and Rossi, 2021). However, repeated applications of fungicides are cost-ineffective and increase the chances of developing fungicide resistance (Corkley et al., 2022). This has been realized in Canada where several *A. rabiei* isolates have developed resistance to prothioconazole and thiabendazole (Wise et al., 2009), azoxystrobin and pyraclostrobin (Delgado et al., 2013; Ahmed et al., 2014; Owati et al., 2017).

The host resistance has been exploited as an environment-friendly management option to control AB disease. However, the development of chickpea cultivars with high levels of resistance to AB has been hampered largely due to a lack of resistant sources within the primary and secondary gene pools (Gayacharan et al., 2020; Newman et al., 2021; Deokar et al., 2024). Complete resistance against *A. rabiei* has not been observed in chickpea germplasm (Jayakumar et al., 2005). Sources with partial or incomplete resistance to AB have been identified and used successfully in different breeding programs to develop moderately resistant cultivars (Raman et al., 2022; Deokar et al., 2024). However, the recent changes in the aggressiveness of the *A. rabiei* pathotypes have eroded the resistance to this disease in commercial chickpea cultivars (Gayacharan et al., 2020; Bar et al., 2021; Raman et al., 2022). The pathogenicity of the Australian isolates is gradually increasing, and the majority of the commercial cultivars with resistant/moderately resistant ratings have become moderately susceptible to very susceptible (Raman et al., 2022). This erosion of resistance poses a constant challenge to developing cultivars with durable resistance to diverse and aggressive pathogen populations.

Plants employ multi-layered defense mechanisms involving constitutive (preformed) and induced (in response to invasion by pathogens) immune systems, including alteration of plant signaling system, and changes in primary and secondary metabolites to combat the persistent battle with phytopathogens (Nehela et al., 2023). Several studies have identified factors including preformed (structural components and secondary metabolites) and induced components of resistance comprising cross-linking of cell walls mediated by hydrogen peroxide, production of pathogenesis-related (PR) proteins (chitinase, β-1,3-glucanase, and thaumatin-like proteins), phytoalexins accumulation, and glutathione *S*-transferase-mediated detoxification associated with resistance against *A. rabiei* in chickpea (reviewed by Jayakumar et al., 2005). Further, the involvement of signaling molecules such as abscisic acid (ABA), ethylene, jasmonic acid (JA), methyl jasmonate, and salicylic acid (SA) in the regulation of defense have been documented in chickpea (Rea et al., 2002; Cho and Muehlbauer, 2004). However, the expression of these induced defense responses did not correlate with pathotype-specific resistance thus the results of these studies need to be validated against the new aggressive isolates using comprehensive approaches.

The necrotrophic pathogens actively suppress, manipulate, and/or evade host defenses during infection via strategies such as inhibition of elicitor-induced defenses, detoxification of plant defense compounds, manipulation of normal physiological processes, and alteration of plant signaling system for successful infection and survival (Laluk and Mengiste, 2010). This complexity in defense response against necrotrophic pathogens complicates the genetic dissection of resistance/susceptibility mechanisms. The understanding of virulence and suppression of host defenses recruited by necrotrophs during host-pathogen interaction and the variation in host resistance/susceptibility mechanisms are important to design strategies for developing resilient cultivars and future-proof crop production (Laluk and Mengiste, 2010).

Therefore, the main objective of this study was to explore the chickpea-*A. rabiei* interaction at the metabolite level to (i) develop an understanding of the host (resistant vs susceptible) metabolites and their role for and against *A. rabiei* during the early stages of infection, and (ii) identify metabolic pathways associated with resistance/susceptibility. We used an untargeted metabolomics approach to investigate the metabolite profile of two Australian accessions, moderately resistant breeding line CICA1841, and a highly susceptible cultivar Kyabra, in response to one of the most aggressive Australian *A. rabiei* isolates; TR9571 representing Pathogenicity group 5 (Bar et al., 2021; Raman et al., 2022).

## Materials and methods

### Plant material

Two accessions; Kyabra and CICA1841, from the CBA (Chickpea Breeding Australia) were selected for this experiment. Kyabra was released in 2005 and is one of the highest-yielding Australian chickpea cultivars with exceptional seed quality but is highly susceptible to AB. CICA1841 is an Australian breeding line developed by Dr. Kristy Hobson, NSW DPI, Tamworth, and has a moderately resistant rating to AB compared to current Australian cultivars (moderately susceptible to very susceptible) (Raman et al., 2022).

### Fungal material and inoculum preparation

One of the most aggressive *A. rabiei* isolates, TR9571 (collected from Gurley, NSW, Australia) was used in this study (https://shinotate.pp18000.cloud.edu.au/shiny/Asco_dashboard/). This isolate was identified as one of the most aggressive isolates (Pathogenicity Group 5) and causes high disease on previously resistant/moderately resistant commercial Australian cultivars (PBA HatTrick, PBA Seamer, and Genesis090) as well as one of the most resistant Syrian landraces (ICC3996) (Bar et al., 2021).

The TR9571 isolate was grown on potato dextrose agar plates. Conidia were harvested by pouring a small amount of sterile water into the plates and gently scraping the spores using a sterile loop. The spore suspension was filtered through a sterile muslin cloth to remove the agar and spore concentration was determined using a hemocytometer. A working concentration of 10^6^ conidia/ml was prepared for inoculations.

### Infection procedure

The experiment was conducted using pots on two benches in an environment-controlled growth chamber at Wagga Wagga Agricultural Institute, Wagga Wagga, NSW, Australia. The experimental design consists of 12 treatments, including two accessions: Kyabra and CICA1841, two infection levels: control and infected, and three sampling times of 24 hr, 48 hr, and 96 hr after infection (hpi). Each treatment consisted of a pot containing 5 plants and there were four biological replicates per treatment, totaling 48 pots [(2 accessions × 2 infection levels (infected and control) × 3 sampling times (24, 48, 96 hpi) × 4 replications)]. Treatments were randomly allocated to positions on the benches under a randomized complete block design in a 4 × 12 array of pots with a 2 × 6 array per replication.

Plants were grown from seed sown in each pot and maintained at a 21°C day/night cycle with a 16/8 hr light regime. At two weeks, plants designated in the infected group were inoculated by spraying the spore suspension (10^6^/ml) until run-off. Control plants were sprayed with sterile water. All pots were covered with plastic domes to maintain humidity for spore germination and initiation of infection. Each plant was scored for disease severity 10 days after inoculation using an ordinal scale of 0-9 (Raman et al., 2022). For metabolomic analysis, aerial tissues (leaf and stem) were collected from the pots designated for each time point: 24-, 48-, and 96 hpi. Each sample represented pooled aerial tissue from the five plants in each pot. Samples were flash-frozen in liquid nitrogen and stored at -80°C. All 48 samples were prepared for LC-MS.

### Liquid chromatography-mass spectrometry analysis

#### Sample preparation

Leaf samples were ground with liquid nitrogen and dried in the freeze dryer for 72 hours. For non-targeted LC-MS, 50 mg of lyophilized powder sample was added into a tube with 800 μL of 80% methanol and homogenized for 90 s, followed by sonication for 30 min at 4°C. Then all samples were kept at -40°C for 1 h. After that, samples were vortexed for 30 s, kept for 0.5 h, and centrifuged at 12000 rpm and 4°C for 15 mins. Finally, 200 μL of supernatant and 5 μL of DL-o-Chlorophenylalanine (140 ug/mL) were transferred to the vial for LC-MS analysis. Separation was performed by Ultimate 3000LC combined with Q Exactive MS (Thermo) and screened with ESI-MS.

The LC system comprised of an ACQUITY UPLC HSS T3 (100×2.1mm, 1.8 μm) with Ultimate 3000LC. The mobile phase was composed of solvent A (0.05% formic acid-water) and solvent B (acetonitrile) with a gradient elution (0-1.0 min, 5% A; 1.0-16.0 min, 5-95% A; 16.0-18.0 min, 95% A; 18.0-19.0 min, 95-5% A; 19.0-20.0 min, 5% A). The flow rate of the mobile phase was 0.3 mL min-1. The column temperature was maintained at 40°C, and the sample manager temperature was set at 4°C. Mass spectrometry parameters in ESI+ and ESI- mode are listed as follows: ESI+: Heater Temp 300°C; Sheath Gas Flow rate, 45arb; Aux Gas Flow Rate, 15arb; Sweep Gas Flow Rate, 1arb; spray voltage, 3.0KV; Capillary Temp, 350°C; S-Lens RF Level, 30%. ESI-: Heater Temp 300°C, Sheath Gas Flow rate, 45arb; Aux Gas Flow Rate, 15arb; Sweep Gas Flow Rate, 1arb; spray voltage, 3.2KV; Capillary Temp,350°C; S-Lens RF Level, 60%.

Quality control (QC) samples were used to assess the reproducibility and reliability of the LC-MS system. The QC samples were run in positive and negative modes at regular intervals throughout the entire sequence.

### Data processing and analysis

Data was obtained in positive and negative electrospray ionization (ESI) modes. The raw data generated were processed for noise filtering, standardization, and normalization before analysis in Thermo Q Exactive. The output from positive and negative ions was saved as Excel tables. The compounds were annotated by comparing the relative molecular mass with the theoretical mass using Thermo Scientific™ Compound Discoverer™ software. The sample preparation and LC-MS analysis were performed by Creative Proteomics (https://www.creative-proteomics.com).

### Statistical analysis

#### *A. rabiei* infection and symptoms development

A linear model was used to describe variation in mean disease score per infected pot in terms of accession, time, and their interaction, plus the replicate blocks. An analysis of variance was derived from the linear model. The model was used to estimate the mean disease severity score for each accession at three sampling times, along with standard errors. The disease severity scores were zero for all the control samples of both accessions, so the data of the control samples was not included in the statistical analysis for disease severity scores. All analyses and graphical presentations were conducted in the R environment (R Core Team, 2023) with the use of the emmeans package (Lenth, 2023).

### Metabolite data analysis

Variation in the accumulation of each metabolite within the two ionization modes was analyzed similarly to the disease scores but with the inclusion of infection level and all interactions. The model was used to estimate means, standard error (SE), and confidence interval (CI) of all metabolites (12 groups; Control CICA1841, Infect CICA1841, Control Kyabra, and Infect Kyabra at 24-,48-,96 hpi). Pairwise differences between mean responses to treatment for all metabolites were calculated and assessed by t-test defined as the ratio (t ratio) of the estimated difference to the standard error. Based on the comparisons metabolites were classed into three categories: (i) discriminatory constitutive metabolites exhibiting a significant (*p*<0.05) varietal effect in the control group (Control CICA1841 - Control Kyabra) at each time point, (ii) altered metabolites with a significant (p<0.05) contrast between infected and control plants within each accession at each time point (Infect Kyabra - Control Kyabra, Infect CICA1841 - Control CICA1841), and (iii) differentially altered metabolites with a significant (*p*<0.05) contrast between infected plants of varieties (Infect CICA1841 – Infect Kyabra). The t ratio was used as a metric for identifying and ranking the most strongly constitutive and altered metabolites. The sign of a t ratio indicates the direction of the difference in samples.

### Annotation of metabolites and metabolic pathway analysis

All constitutive and altered metabolites were annotated using KEGG (Kyoto Encyclopedia of Genes and Genomes), and HMDB (Human Metabolome Database). The pathway analysis on significantly altered metabolites was performed using the Pathway Analysis module of MetaboAnalyst 5.0 (https://genap.metaboanalyst.ca/MetaboAnalyst/). A list of compound/metabolite names was used as input and *Arabidopsis thaliana* was chosen as the pathway library. The hypergeometric test was selected as the over-representation analysis method. Results from the over-representation analysis were used for generating bubble plots using SR plot online (https://www.bioinformatics.com.cn/en?keywords=bubble). The pathways with *p*-values (<0.05), false discovery rate (FDR) <0.05, and pathway impact (>0.1) were considered candidates related to chickpea-*A. rabiei* interaction. The pathway collage was obtained by overlaying twenty-seven differentially altered metabolites and their respective t ratio on the cellular overview/omics viewer from Plant Metabolic Network (PMN) plantcyc using *Cicer arietinum* metabolic background (https://pmn.plantcyc.org/overviewsWeb/celOv.shtml?orgid=CARIETINUM_ICC4958). The metabolite-metabolite interaction network was performed using MetaboAnalsyt 5.0 to identify the interactions of the twenty-seven differentially altered metabolites and a minimum network filter was used to construct the network (https://genap.metaboanalyst.ca/MetaboAnalyst/).

## Results

### *Ascochyta rabiei* infection and symptom development

**Figure 1:**
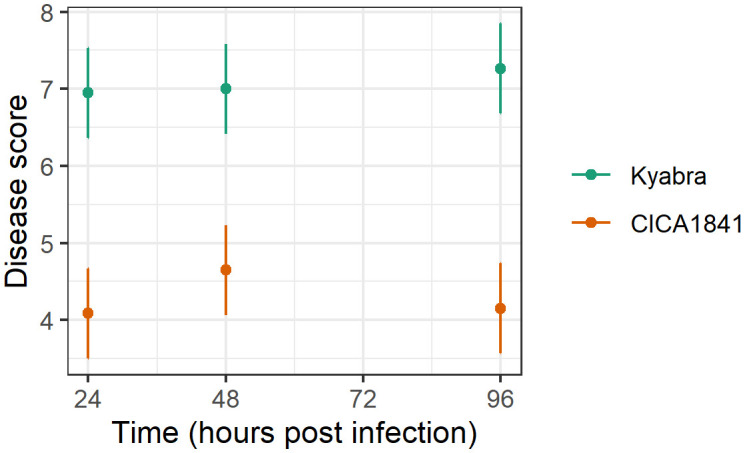
Average disease score (points) and 95% confidence intervals (lines) of two accessions against TR9571 isolate in the samples collected over time (24, 48, 96 hpi) for metabolite analysis. Kyabra had a higher disease rating compared to CICA1841.

The development of symptoms in the inoculated plants was monitored weekly for 3 weeks post-inoculation. Disease evaluation was carried out as described previously (Raman et al., 2022). Significant differences in Ascochyta blight disease scores were observed between the two accessions (**Supplementary Table 1**). Increased levels of disease (p<0.001) were observed in the susceptible cultivar Kyabra (average score ± se = 7.1 ± 0.2) compared to the moderately resistant breeding line CICA1841 (average score ± se = 4.3 ± 0.2) against isolate TR9571 (**Figure 1**). The effect of time was not statistically significant (p>0.05). The control plants of both accessions were healthy.

### Metabolomic profiling of moderately resistant and highly susceptible accessions based on LC-MS

Two accessions with variable responses to *A. rabiei* isolate; TR9571 were selected to elucidate the resistance/susceptibility mechanism at a metabolite level. The infected and control samples of both accessions were evaluated at three time points; 24 hpi, 48 hpi, and 96 hpi to identify differential metabolites. Aerial tissue extracts were analyzed using untargeted metabolomics using LC-MS and data was acquired in both positive and negative electrospray ionization (ESI) modes. The results show moderate metabolite diversity in the two chickpea accessions in response to infection. In total, 236 (negative ESI) and 224 (positive ESI) metabolites were identified (137) were common across two modes. The mass abundance (raw data) for all metabolites in each of the 48 samples among the 12 experimental groups (two accessions x two treatments x three time points) are presented in **Supplementary Tables 2A, B**. The analyzed means (ES), standard error (SE), and confidence interval (CI) of all metabolites (12 groups) are provided in **Supplementary Tables 3A, B**.

### Discriminatory metabolites in moderately tolerant and susceptible accessions without infection (constitutive)

**Figure 2:**
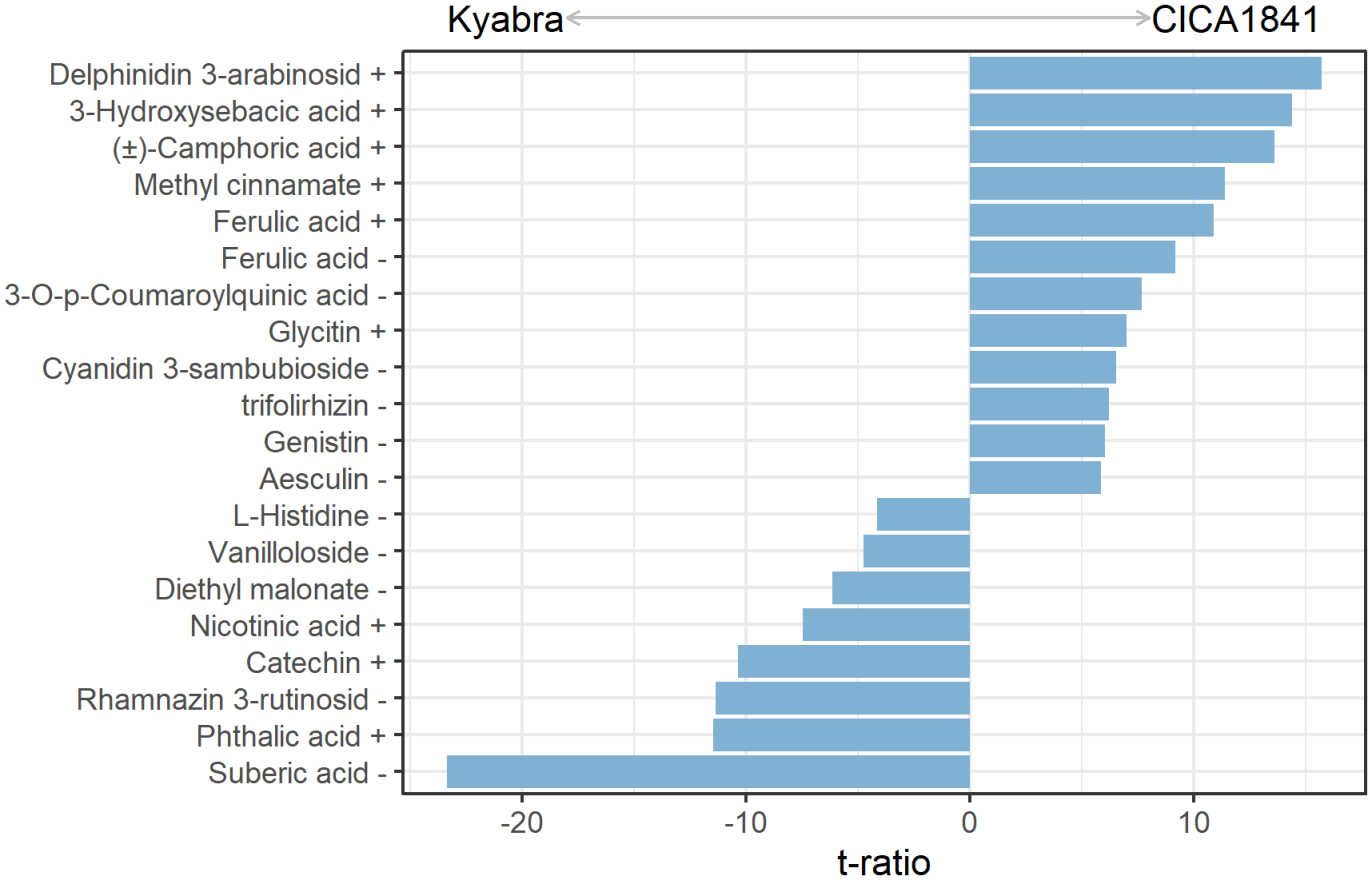
Constitutive metabolites with significant differences in mass abundance between control samples of CICA1841 and Kyabra. Metabolites with positive t ratio had higher mass abundance in CICA1841 and metabolites with negative t ratio had higher mass abundance in Kyabra. The + and – sign after the metabolite indicates their detection in positive and negative ion mode respectively.

A total of 19 metabolites differed significantly in moderately resistant and highly susceptible accessions without infection (control samples) (**Figure 2**). Out of 19, eleven metabolites show a significantly higher mass abundance in the moderately resistant accession CICA1841 as compared to Kyabra (t ratio = 5.9 to 15.7, *p*-value = < 1.5E^-06^), for example, delphinidin 3-arabinoside, 3-hydroxysebacic acid, (±)-camphoric acid, methyl cinnamate, and ferulic acid) (**Figure 2**; **Supplementary Table 4A**). In comparison, suberic acid, phthalic acid, rhamnazin 3-rutinoside, catechin, and nicotinic acid (t ratio = -4.2 to -23.4, *p*-value = < 2.2E^-04^) were in higher abundance in the susceptible accession (**Figure 2**; **Supplementary Table 4A**).

### Altered metabolites in moderately tolerant and susceptible accessions after infection

We observed differences in metabolite accumulation in the infected and control samples of two accessions. The list of metabolites altered in CICA1841 (Infect CICA1841 - Control CICA1841) and Kyabra (Infect Kyabra - Control Kyabra) at all three time points after infection is provided in **Supplementary Tables 4B, C** respectively. The *p*-value cut-off of < 0.01 was applied to select the altered metabolites for both accessions. The susceptible accession, Kyabra, exhibited a higher number of altered metabolites and higher values of the t ratio (change in abundance after infection) compared to the moderately resistant CICA1841 over four days (**Supplementary Tables 4B, C**).

**Figure 3:**
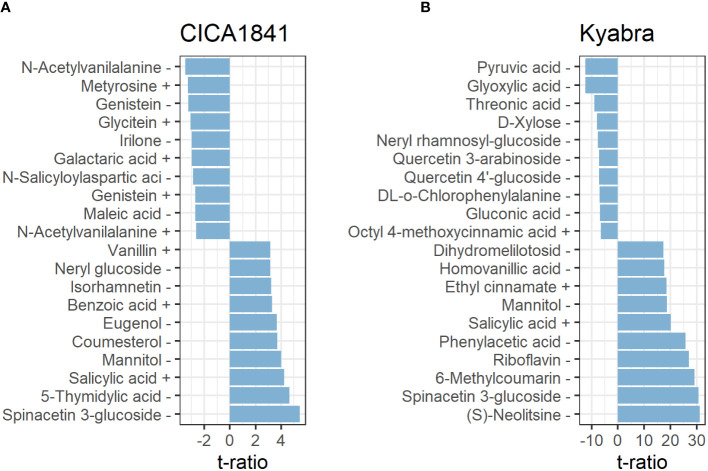
Metabolites with the ten most positive and negative effects in CICA1841 **(A)** and Kyabra **(B)** at 96 hpi. Metabolites showing a positive t ratio had mass abundance increased in infected plants compared to the control while negative values indicate a reduction in mass abundance in infected plants compared to the control. The + and – sign after the metabolite indicate their detection in positive and negative ion mode respectively.

In total, twenty-seven metabolites were altered at all three time points in CICA1841. Within 24 hpi, five metabolites, eg., pantothenic acid, mevalonic acid, and quercetin were significantly altered (induced) (t ratio = -2.7 to 3.3, *p*-value< 0.01), though their mass abundance was not significantly different as compared to the control samples when collected at 48 hpi, and 96 hpi. However, at 96 hpi, nineteen metabolites were significantly altered (13 induced and 6 suppressed) in CICA1841, eg., spinacetin 3-glucoside, 5-thymidylic acid, salicylic acid, mannitol, benzoic acid were induced (t ratio = +2.6 to 5.5, *p-value <*0.01), and N-acetylvanilalanine, genistein, irilone, and maleic acid were suppressed (t ratio = -2.7 to 3.5, *p*-value <0.01) after infection with *A. rabiei* (**Figure 3A**; **Supplementary Table 4B**).

Interestingly, Kyabra showed a distinct response compared to CICA1841. A total of 170 metabolites were altered in the susceptible accession Kyabra at all three time points (**Supplementary Table 4C**). Only five metabolites were altered (induced) within 24 hpi (2,3-Di-O-methylellagic acid, 2-Isopropylmalic acid, gallic acid, itaconic acid, and cyclic AMP), though within 48 hpi, 78 metabolites were significantly altered, of these 38 were induced (t ratio = 2.6 to 11.9, *p*-value< 0.01) and 40 were suppressed (t ratio = -2.6 to -6.3, *p*-value < 0.01) indicating some early response to infection. For example, the mass abundance of naringenin, riboflavin, phloretin, benzoic acid, and salicylic acid, were significantly induced, and pyruvic acid, L-methionine, and D-fructose were suppressed. At 96 hpi, when the fungus had established itself within the plant, many metabolites (166) were significantly altered in the susceptible accession; of these 64 were induced (t ratio = 2.6 to 31.1, *p-value ≤*0.01), and 102 were suppressed (t ratio ≥ 2.6 to 12.5, *p-value ≤*0.01) in response to *A. rabiei* (**Supplementary Table 4C**). Some of the significantly altered compounds in CICA1841 and Kyabra in response to *A. rabiei* at 96 hpi are shown in **Figures 3A, B**.

### Differentially altered metabolites in moderately resistant BL (CICA1841) and susceptible cultivar (Kyabra) in response to *A. rabiei*

**Figure 4:**
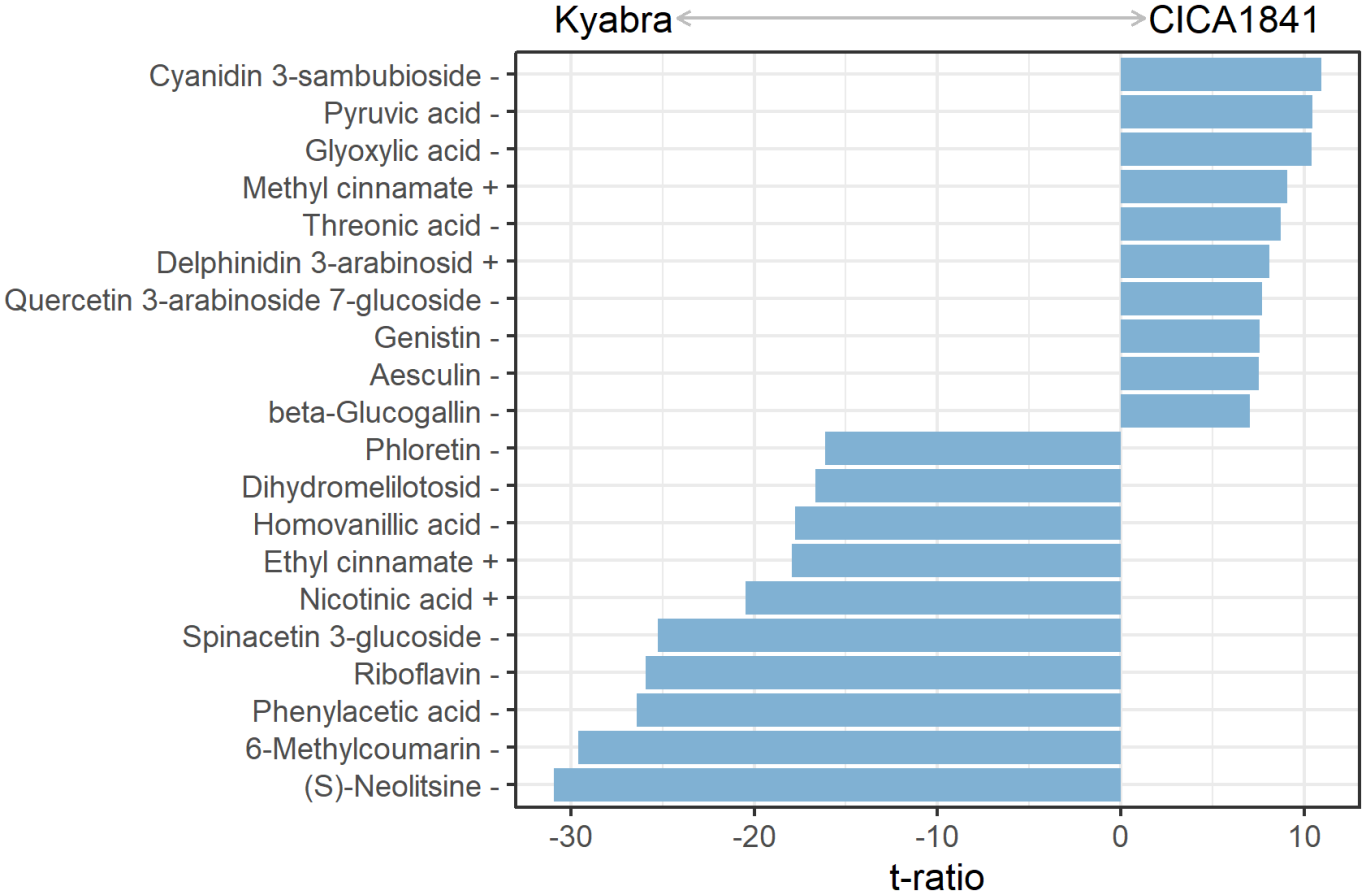
Discriminatory metabolites with the ten most positive and negative varietal effects on the mass abundance in infected plants at 96 hpi. Positive values had significantly higher mass abundance in CICA1841 than in Kyabra (p<0.05). The + and – sign after the metabolite indicate their detection in positive and negative ion mode respectively.

The levels of metabolites were compared among infected samples of moderately resistant and susceptible accessions (Infect CICA1841 - Infect Kyabra) at all three time points. In total, 184 differentially altered metabolites were identified (**Supplementary Table 4D**). During the early stages, 29 differential metabolites (18 high abundance and 11 low abundance) and 76 (50 high abundance and 26 low abundance) metabolites were identified at 24 hpi and 48 hpi, respectively. Some of the most significantly altered metabolites at 96 hpi are shown in **Figure 4**.

In comparison, 147 metabolites were differentially altered in the aerial tissue of the susceptible cultivar Kyabra compared with CICA1841 after fungus colonization at 96 hpi. Of these, fifty-nine metabolites were significantly induced, and eighty-eight were suppressed in Kyabra in comparison with CICA1841. The differentially altered compounds included organic acids, benzenoids, hormones, pteridines, sugar, sugar alcohols, and flavonoids. For example, the accumulation of phenylacetic acid, riboflavin, salicylic acid (SA), nicotinic acid, and mannitol was upregulated after infection with *A. rabiei* in the susceptible cultivar, Kyabra, indicating high levels of these compounds are probably required for pathogenicity. Noticeably, the endogenous levels of Nicotinic acid were high in Kyabra compared with CICA1841 (t ratio = -7.5) without infection, and they were further increased in Kyabra at 96 hpi after infection (t ratio = -20.5) (**Figures 2**, **4**; **Supplementary Table 4D**).

The eighty-eight metabolites suppressed in Kyabra compared to the moderately resistant accession CICA1841 at 96 hpi included pyruvic acid, glyoxylic acid, threonic acid, D-xylose, methyl jasmonate, and rutin, indicating that *A. rabiei* has a stronger inhibitory effect on these compounds for its survival in the host. A higher number of primary metabolites were suppressed in the susceptible accession after infection, indicating the role of primary metabolism in susceptibility to *A. rabiei*.

**Figure 5:**
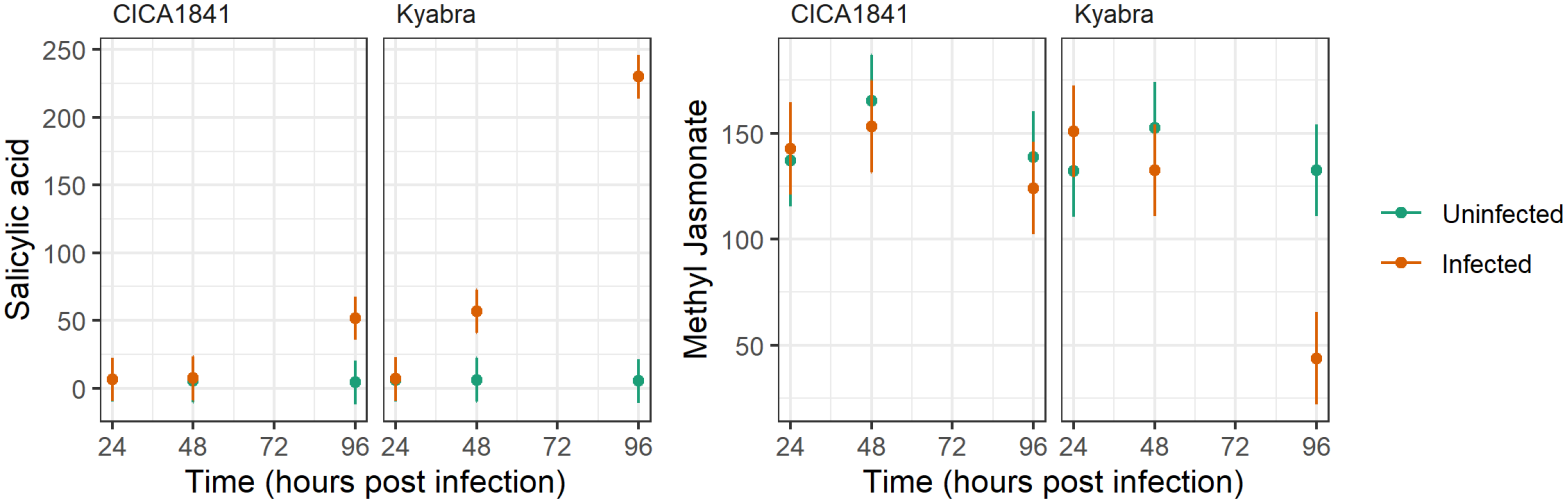
Average mass abundance (points) and 95% confidence intervals (lines) for Salicylic acid and Methyl Jasmonate in response to accession, time, and infection with *A. rabiei* isolate TR9571.

Interestingly, endogenous levels of salicylic acid and methyl jasmonate did not differ significantly in the two accessions, though they were altered after the infection; salicylic acid was significantly induced in Kyabra, and methyl jasmonate was suppressed in Kyabra after infection at 96 hpi (**Figure 5**). These results indicate the role of SA and JA pathways in chickpea-*A. rabiei* interaction. Overall, our results highlight the manipulation of endogenous levels of metabolites in chickpeas by *A. rabiei*.

### Pathway analysis of metabolites associated with chickpea-*A. rabiei* interaction

The differentially altered metabolites (induced or suppressed) after infection at 48- and 96 hpi were used for pathway enrichment analysis. The results of pathway analysis using the KEGG database suggested possible biological pathways activated in chickpeas in response to *A. rabiei*. The pathway results are presented in **Supplementary Table 5A**.

**Figure 6:**
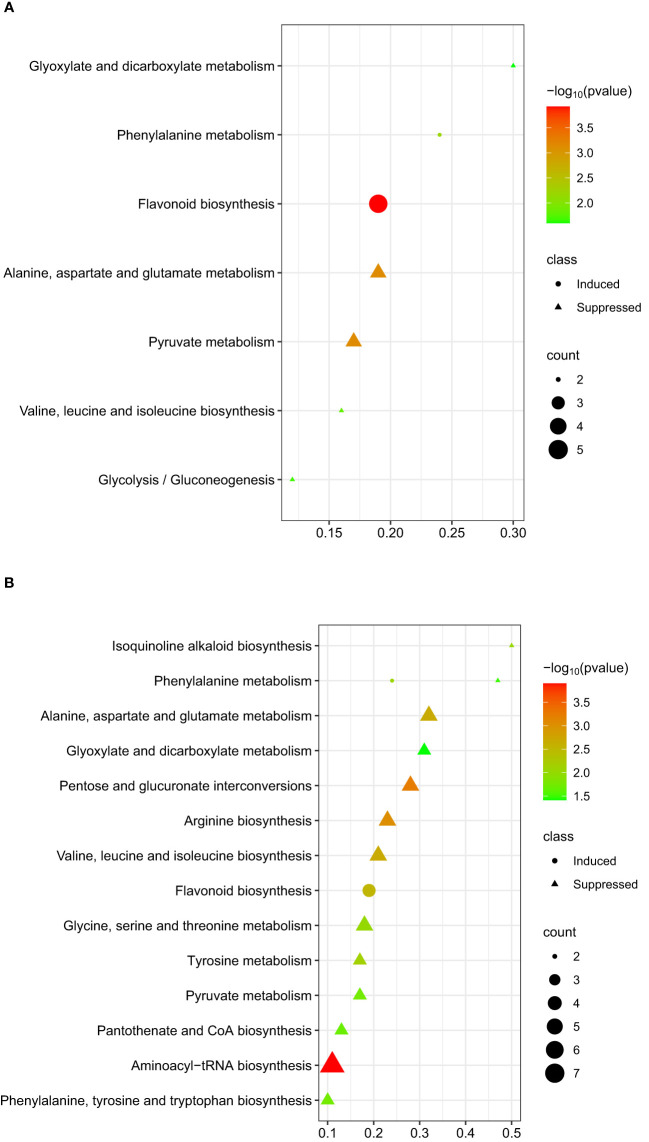
Graphical summary plot of metabolic pathway analysis using Metaboanalyst 5.0. The plot shows metabolic pathways in chickpeas in response to *A*. *rabiei* (y-axis); The x-axis represents the pathway impact value computed from pathway topological analysis, *P*-value is obtained from pathway enrichment analysis; The color and size of each circle correspond to its *p*-value and metabolite count, respectively; The pathways that were most significantly changed are characterized by both a high -log(*p*) value and high impact value; **(A)** plot showing possible pathways involved by taking metabolites induced or suppressed at 48 hpi; **(B)** plot showing possible pathways involved by taking metabolites induced or suppressed at 96 hpi.

The flavonoid pathway was the most significant pathway identified for the metabolites that were differentially induced within 48 hpi (**Figure 6A**). For the differentially suppressed metabolites at 48 hpi, two candidate metabolic pathways, pyruvate metabolism, and alanine, aspartate, and glutamate metabolism were identified (**Figure 6A**). At 96 hpi, five metabolic pathways; aminoacyl-tRNA biosynthesis, pentose and glucuronate interconversions, arginine biosynthesis, alanine, aspartate, and glutamate metabolism, and valine, leucine, and isoleucine biosynthesis were significantly correlated with differentially suppressed metabolites (**Figure 6B**).

**Figure 7:**
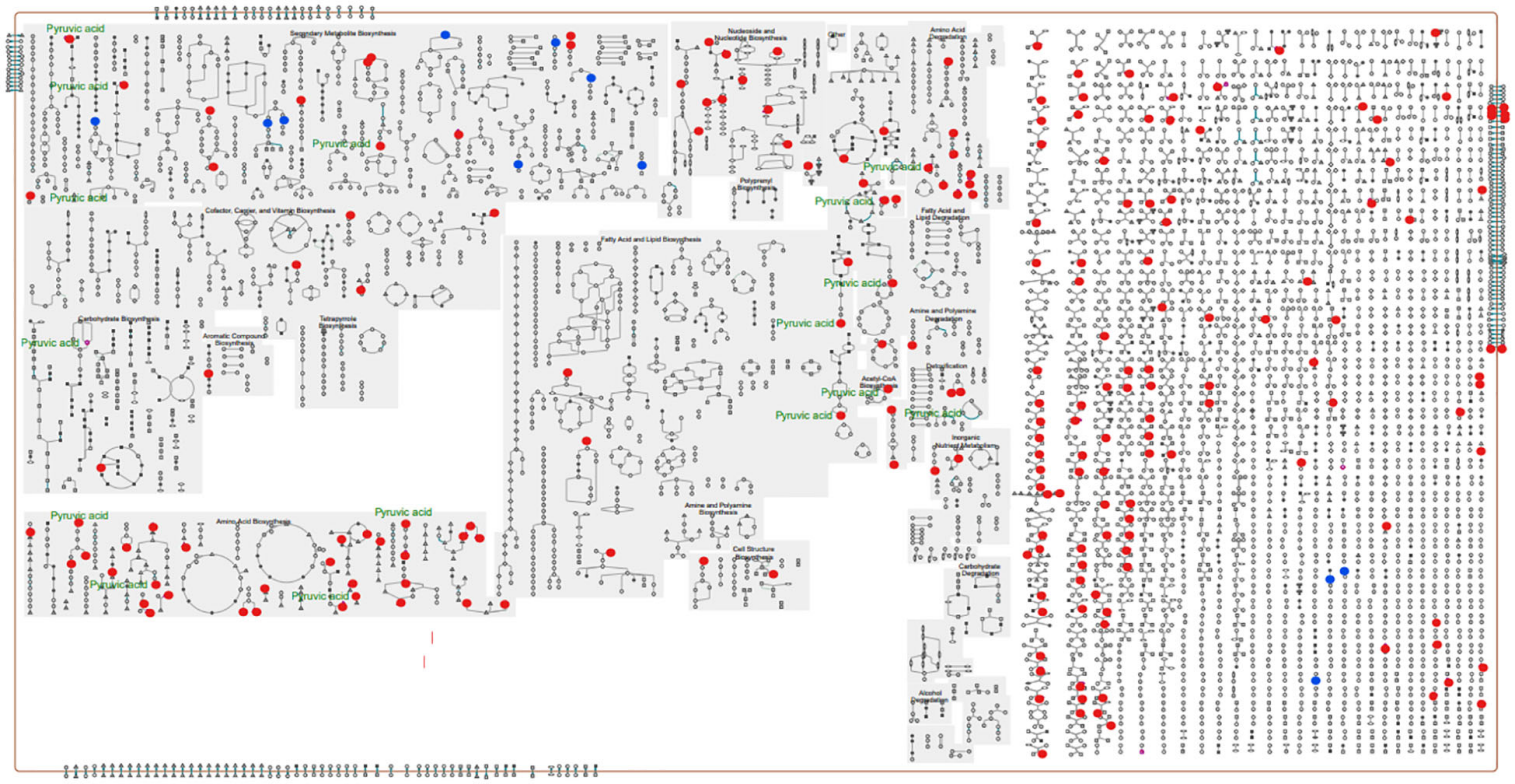
Pathway collage diagram generated with Pathway Collage module under Plant Metabolic Network (PMN) and *C. arietinum* database showing functionally important metabolic pathways (identified using MetaboAnalyst 5.0). The upregulated (blue) and downregulated (red) discriminatory annotated metabolites identified at 96 hpi are shown along with their involvement in metabolic pathways in the ‘cellular overview’ of the pathway collage diagram.

**Figure 8:**
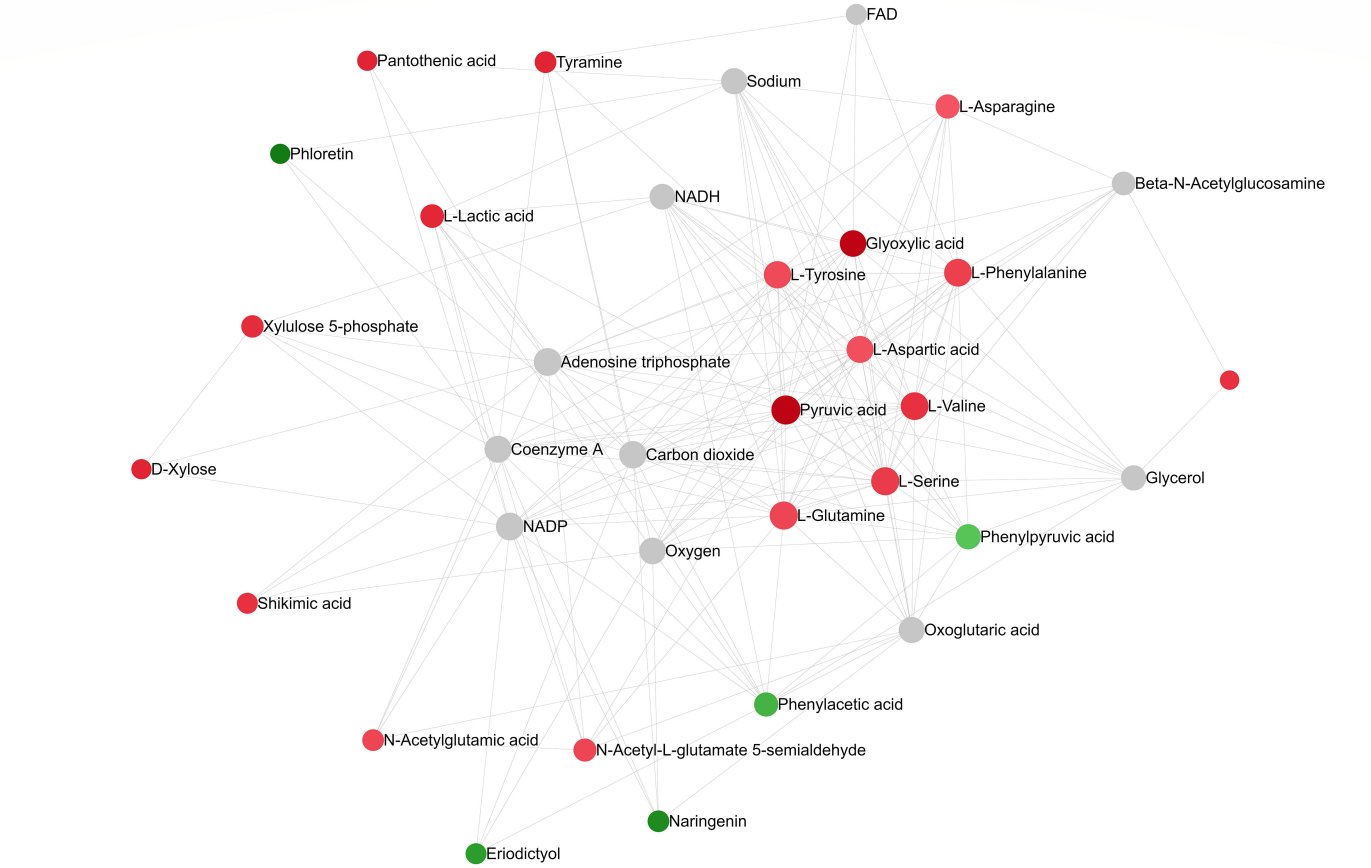
Metabolite-metabolite interaction network post *A. rabiei* infection (96 hrs) in chickpea. The metabolites represent functionally important metabolic pathways (identified using MetaboAnalyst 5.0). The metabolites are represented as nodes (circles). The red color represents upregulated and green for downregulated discriminatory metabolites. Compounds with higher betweenness centrality are highlighted in lighter shades of red and green. The size of the node indicates node degree (number of connections to other nodes).

A pathway collage generated using the twenty-seven differentially altered metabolites (96 hpi) (identified using MetaboAnalyst 5.0; **Supplementary Table 5B**) and the *C. arietinum* database highlighted different chickpea metabolic pathways involving metabolites that altered in response to *A. rabiei* infection (**Figure 7**). The interactive collage provided all occurrences of a particular metabolite in all metabolic pathways and its relationship with other metabolites in the pathways. For example, pyruvic acid, one of the downregulated metabolites in the susceptible cultivar was in multiple interconnecting metabolic pathways suggesting that the role of pyruvic acid is critical in chickpea-*A.rabiei* interaction (**Figure 7**). Further, the network analysis pin-pointed the metabolite-metabolite interactions of twenty-seven altered metabolites from fifteen metabolic pathways (*p*-value = <0.05; pathway impact = >0.1) and is presented in **Figure 8**. Pyruvic acid, L-glutamine, L-serine, L-aspartic acid, and L-tyrosine were the key metabolites and showed maximum interactions with other metabolites indicating their functional relationship in the most significant metabolic pathways associated with chickpea – *A. rabiei* interaction (**Supplementary Table 6**).

## Discussion

In this study, we performed metabolite profiling of two chickpea accessions with contrasting reactions to *A. rabiei* isolate TR9571 to identify metabolite changes after infection using a non-targeted LC-MS approach. The analysis showed that nineteen metabolites (constitutive) could discriminate two chickpea accessions as their levels were significantly different in the control samples of Kyabra and CICA1841. High levels of ferulic acid (phenolic compound) in plants were observed in the CICA1841 compared to Kyabra in the control samples. Several phenolic compounds are regarded as pre-infection inhibitors, providing plants with a certain degree of basic resistance against pathogenic microorganisms (Vagiri et al., 2017).

The high abundance of defense-related metabolites such as ferulic acid may act as a pre-infection inhibitor to resist *A. rabiei* infection in chickpea. Ferulic acid has been shown to have a strong inhibitory effect against tobacco black shank disease (Zhang et al., 2020) and was also found to significantly increase total phenol and flavonoid accumulations in apples after infection with grey mold (He et al., 2019). It has been reported that ferulic acid, an intermediate of the phenylpropanoid pathway binds to lignin, polysaccharides, and structural proteins of grass cell walls cross-linking these components (Reem et al., 2016). Interestingly, the study further demonstrated that the reduction of ferulic acid content negatively affected plant resistance to biotic stress, most likely due to compromised cell wall integrity (CWI). We speculated that low ferulic acid in Kyabra is likely compromising the CWI, and in turn, making it very susceptible to fungus invasion.

The high mass abundance of catechin in the susceptible cultivar is likely favoring the *A. rabiei* growth and infection. The dual role of catechin, a phenolic compound in defense and susceptibility to pathogens has been reported. Maia et al. (2020) hypothesized that some fungal/oomycetes pathogens of grapevine may be using catechins as a carbon source for growth and successful infection. Both the catechin and epicatechin are possible susceptibility biomarkers as the leaves of the susceptible grapevine cultivars had higher levels of catechin (Maia et al., 2020).

It is worth mentioning that six putative resistance-associated metabolites (high in CICA1841) (methyl cinnamate, ferulic acid, 3-O-p-coumaroylquinic acid, glycitin, cyanidin 3-sambubioside, and genistin) and two susceptibility-associated metabolites (rhamnazin 3-rutinoside, and catechin) belong to the phenylpropanoid pathway. The genes involved in the phenylpropanoid pathway have been associated with resistance to AB in chickpea (Cho et al., 2005; Jaiswal et al., 2012; Chandel et al., 2023). More recently, Singh et al. (2023b) established that *A. rabiei* effector protein, ArPEC25 interrupts phenylpropanoid biosynthesis by interrupting lignification of the cell wall in chickpea. The cell wall is the most important barrier that defends plants against pathogens and its integrity largely determines the fate of the host-pathogen interaction. Once the cell wall is compromised, the necrotrophs secrete disease-causing agents including phytotoxins, and cell wall degrading enzymes (CWDEs) to destroy host tissue and release nutrients that promote colonization (Bellincampi et al., 2014; Zhang et al., 2022). Further work is required to validate the results and develop bioassays for positive (resistant) and negative (susceptible) selection of the breeding germplasm based on the endogenous level of these metabolites/biomarkers in accessions with varying degrees of resistance/susceptibility.

We observed a low number of differential metabolites which had their levels either induced or suppressed in CICA1841, a possible explanation for the low level of disease as compared to Kyabra. It has been reported that *A. rabiei* spores germinate within 12-24 h on landing on the host surface (Pandey et al., 1987). Also, the time for spore germination and appressorium formation differs for resistant and susceptible accessions of chickpea (Bar et al., 2021). For example, more aggressive *A. rabiei* isolates generally develop appressoria by 30–40 hpi on the susceptible host, while appressorium formation is delayed to 60–70 hpi, and also only a few appressoria are formed in the resistant host. This could explain the low level of metabolic change in CICA1841 after infection. The mass abundance of two metabolites, pantothenic acid, and quercetin was significantly altered (induced) at 24 hpi in the resistant accession, and their mass abundance was suppressed in Kyabra at 96 hpi indicating their role in providing some resistance during an early phase of the *A. rabiei* infection. Interestingly, quercetin is a sub-class of flavonoid, and its antioxidant and reactive oxygen species (ROS) modulating properties potentially provide tolerance to biotic and abiotic stresses. Recently, Wang et al. (2022) reported the efficacy of quercetin nanoliposomes by using a nano-delivery system for the control of plant virus diseases in tobacco. Pantothenic acid (vitamin B5) is an essential compound in plant metabolism and its upregulation is an indication of stress adaptation in plants (Singh et al., 2023a). Previously, Jayakumar et al. (2005) have suggested that a higher amount of phytoalexins in resistant chickpea cultivars may have delayed disease development and reduced disease severity.

A total of 184 differential metabolites were identified in the aerial tissue samples at three time points indicating that the fungus is manipulating the level of host metabolites. Among the fifty-nine up-regulated discriminant metabolites at 96 hpi, flavonoids (7), carboxylic acids and derivatives (7), and isoflavonoids (5) were the major classes. The increased levels may indicate their role in promoting fungus growth and proliferation resulting in enhanced susceptibility. Many pathogens employ effector proteins within plant cells upon infection to suppress plant immunity. These effector proteins hijack plant metabolism resulting in the biosynthesis of metabolites that act as nutrients during fungal colonization (Shao et al., 2021).

While, carboxylic acids and derivatives (17), flavonoids (8), and acyl acids (7) were the major class of eighty-eight down-regulated discriminant metabolites at 96 hpi. The reduced levels after infection may indicate their role in plant growth and development including in disease resistance as reduction of these metabolites is likely facilitating the spread of fungus in Kyabra compared to CICA1841. Buhtz et al. (2015) reported reduced levels of major cell wall components like glucuronic acid and xylose in the presence of soil-borne fungus *Verticillium dahliae* in tomato.

Further, our study showed that *A. rabiei* fungus altered many metabolites which either generate/induce or scavenge ROS to its advantage and establish successful infection and proliferation in the susceptible chickpea cultivar. For example, mannitol (ROS scavenger), nicotinic acid, glutathione (regulate ROS), and riboflavin (induce ROS), were induced in Kyabra in response to the *A. rabiei* infection probably to balance ROS. Glutathione is an essential metabolite and is known to regulate ROS. Both mannitol and glutathione (are necessary for resistance and pathogenesis Calmes et al., 2013; Hernández et al., 2017; Zechmann, 2020; Wangsanut and Pongpom, 2022). Maurya et al. (2020) identified the upregulation of oxidative stress marker genes like glutathione reductase, in *A. rabiei* during oxidative stress (menadione treatment) as compared to control. Maintaining redox homeostasis is important for fungus and the glutathione redox system plays a major role when *A. rabiei* is under oxidative stress (Maurya et al., 2020). The imbalance generated by the reactive oxygen species and scavenging system (oxidative stress) may result in tissue damage of the susceptible cultivar thus making it an easy target. We speculate that the interplay between ROS generating, and scavenging metabolites may be one of the important factors for determining the outcome of the chickpea-*A. rabiei* interaction.

Additionally, in our study, we found altered levels of phytohormones in the infected plants. For example, there was no significant difference in the mass abundance of MeJA in CICA1841 and Kyabra without infection (controls) at three time points, though by 96 hpi after infection it was downregulated significantly in Kyabra (t ratio = 5.32, *p-value* = 7.13E^-06^). Interestingly, the endogenous levels of SA were low and did not differ significantly in the control samples of both resistant and susceptible accessions (**Figure 5**). However, SA levels were significantly upregulated in Kyabra as compared to CICA1841 at 96 hpi after infection with *A. rabiei* (t ratio = -15.97, *p-value* = 4.37E^-17^). Downregulation of MeJA and upregulation of SA in Kyabra after infection might contribute to its susceptibility. The activation of the JA signaling pathway is important for resistance against necrotrophic pathogens generally whereas the SA signaling pathway is mainly activated against biotrophic pathogens (El Oirdi et al., 2011). The role of SA in providing resistance to necrotrophic fungus has been identified in potato (Brouwer et al., 2020). Both SA and JA have been known to act antagonistically and some pathogen exploits this antagonism as a strategy to overcome the plant’s defense system and spread within the host (Klemme et al., 2019). For example, *Botrytis cinerea* produces an exopolysaccharide (an elicitor of the SA pathway) which then antagonizes the JA signaling pathway, allowing the fungus to cause grey mold in tomato (El Oirdi et al., 2011). Our results indicate a possible role of SA and JA in chickpea-*A. rabiei* interaction and warrants further investigation if *A. rabiei* is using a similar strategy as *B. cinerea* to cause AB in chickpea. Recently Chandel et al. (2023) have reported the role of JA, ABA, and GA biosynthesis genes in phytohormone metabolic pathways in determining the resistance or susceptibility to *A. rabiei* in chickpea. Their study concluded that MeJA is associated with resistance and ABA with susceptibility. This finding supports our results that MeJA is likely providing some resistance to *A. rabiei* isolate TR9571. In CICA1841, the mass abundance of MeJA was high in the control samples and remained unchanged after infection (unaffected by *A. rabiei*), though in Kyabra, the levels were significantly low after infection (96 hpi).

### Differential metabolic reconfiguration of host metabolic pathways

The flavonoid biosynthesis pathway was activated during the early phase of infection (48 hpi) by the *A. rabiei.* Flavonoids can control the accumulation of ROS to cause cell death and maintain a crucial balance between ROS generation and antioxidant defense. The flavonoids in this pathway; eriodictyol, naringenin, phloretin, liquiritigenin, and luteolin, were differentially induced and may be providing an ideal environment for invasion by necrotrophic fungus in the susceptible host (Kyabra).

*A. rabiei* also suppressed the compounds that may be associated with growth and development and with resistance. Five pathways were associated with the metabolites that were significantly reduced in Kyabra compared to CICA1841 at 96 hpi; aminoacyl-tRNA biosynthesis, pentose and glucuronate interconversions, arginine biosynthesis, valine, leucine and isoleucine biosynthesis, and alanine, aspartate and glutamate metabolism. Amino acids, tyrosine, and phenylalanine are involved in the synthesis of several secondary metabolites through the phenylpropanoid biosynthesis pathways leading to the accumulation of lignins (Fraser and Chapple, 2011; Parthasarathy et al., 2018; Mashabela et al., 2023). The decreased accumulation of the amino acids in the susceptible accession after infection indicated the disruption of the cell wall components, the first line of defense against pathogens, and depriving the host of nutrients.

The pathway collage and metabolite-metabolite interaction analysis highlighted linkages between significantly altered metabolites and provided an overview of the altered metabolic network. The interaction between amino acids and secondary metabolites indicated their functional role in *A. rabiei* interaction and a diverse response by the host. These results will help to understand and further explore the biological processes and the potential functional relationships between metabolites in determining the outcome (resistance/susceptibility) of the chickpea-*A. rabiei* interaction. Overall, the results of our study indicate a complex interplay between the host and fungus which needs to be further explored to establish the role of compounds in these pathways in pathogenicity and resistance.

## Conclusions

In this study, we profiled metabolomes of two Australian chickpea accessions comprising a moderately resistant breeding line (CICA1841) and a highly susceptible cultivar (Kyabra) in response to *A. rabiei* infection. We have identified putative metabolites associated with resistance and susceptibility to one of the most aggressive Australian *A. rabiei* isolates TR9571. Our results showed that the metabolome of the moderately resistant host did not go through extensive modulation as it was less impaired by fungus and only a few changes related to the defense mechanism occurred at the metabolite level compared to the susceptible cultivar. Overall, our results have provided a starting point for finding functional links between AB disease and metabolic pathways. Further research on integrating the metabolite results with transcriptional and genetic analysis on the same set of samples will elucidate the metabolic pathway interactions and the mechanisms of resistance and susceptibility to Ascochyta blight in chickpea. The prospects of reducing the level of Ascochyta blight in chickpea and increasing crop productivity lie in a multi-level analysis.

## Data Availability

The original contributions presented in the study are included in the article/**Supplementary Material**. Further inquiries can be directed to the corresponding author.
